# Serum thymidine kinase 1 activity as a pharmacodynamic marker of cyclin-dependent kinase 4/6 inhibition in patients with early-stage breast cancer receiving neoadjuvant palbociclib

**DOI:** 10.1186/s13058-017-0913-7

**Published:** 2017-11-21

**Authors:** Nusayba Bagegni, Shana Thomas, Ning Liu, Jingqin Luo, Jeremy Hoog, Donald W. Northfelt, Matthew P. Goetz, Andres Forero, Mattias Bergqvist, Jakob Karen, Magnus Neumüller, Edward M. Suh, Zhanfang Guo, Kiran Vij, Souzan Sanati, Matthew Ellis, Cynthia X. Ma

**Affiliations:** 1 Division of Oncology, Section of Medical Oncology, Department of Medicine, Siteman Cancer Center, Washington University School of Medicine, 660 South Euclid Avenue, St. Louis, MO 63110 USA; 2 Mayo Clinic, Phoenix, AZ 85054 USA; 3 Mayo Clinic, Rochester, MN 55905 USA; 4 University of Alabama at Birmingham, Birmingham, AL 35294 USA; 5 Biovica International AB, Uppsala, Sweden; 6 Pelago Bioscience AB, Solna, Sweden; 7 Baylor College of Medicine, Houston, TX 77030 USA

**Keywords:** Breast cancer, Thymidine kinase, Palbociclib, Anastrozole, Neoadjuvant, Biomarker

## Abstract

**Background:**

Thymidine kinase 1 (TK1) is a cell cycle-regulated enzyme with peak expression in the S phase during DNA synthesis, and it is an attractive biomarker of cell proliferation. Serum TK1 activity has demonstrated prognostic value in patients with early-stage breast cancer. Because cyclin-dependent kinase 4/6 (CDK4/6) inhibitors prevent G_1_/S transition, we hypothesized that serum TK1 could be a biomarker for CDK4/6 inhibitors. We examined the drug-induced change in serum TK1 as well as its correlation with change in tumor Ki-67 levels in patients enrolled in the NeoPalAna trial (ClinicalTrials.gov identifier NCT01723774).

**Methods:**

Patients with clinical stage II/III estrogen receptor-positive (ER+)/HER2-negative breast cancer enrolled in the NeoPalAna trial received an initial 4 weeks of anastrozole, followed by palbociclib on cycle 1, day 1 (C1D1) for four 28-day cycles, unless C1D15 tumor Ki-67 was > 10%, in which case patients went off study owing to inadequate response. Surgery occurred following 3–5 weeks of washout from the last dose of palbociclib, except in eight patients who received palbociclib (cycle 5) continuously until surgery. Serum TK1 activity was determined at baseline, C1D1, C1D15, and time of surgery, and we found that it was correlated with tumor Ki-67 and TK1 messenger RNA (mRNA) levels.

**Results:**

Despite a significant drop in tumor Ki-67 with anastrozole monotherapy, there was no statistically significant change in TK1 activity. However, a striking reduction in TK1 activity was observed 2 weeks after initiation of palbociclib (C1D15), which then rose significantly with palbociclib washout. At C1D15, TK1 activity was below the detection limit (<20 DiviTum units per liter Du/L) in 92% of patients, indicating a profound effect of palbociclib. There was high concordance, at 89.8% (95% CI: 79.2% - 96.2%), between changes in serum TK1 and tumor Ki-67 in the same direction from C1D1 to C1D15 and from C1D15 to surgery time points. The sensitivity and specificity for the tumor Ki-67-based response by palbociclib-induced decrease in serum TK1 were 94.1% (95% CI 86.2% - 100%) and 84% (95% CI 69.6% -98.4%), respectively. The κ-statistic was 0.76 (*p* < 0.001) between TK1 and Ki-67, indicating substantial agreement.

**Conclusions:**

Serum TK1 activity is a promising pharmacodynamic marker of palbociclib in ER+ breast cancer, and its value in predicting response to CDK4/6 inhibitors warrants further investigation.

**Trial registration:**

ClinicalTrials.gov, NCT01723774. Registered on 6 November 2012.

**Electronic supplementary material:**

The online version of this article (doi:10.1186/s13058-017-0913-7) contains supplementary material, which is available to authorized users.

## Background

Cytosolic thymidine kinase 1 (TK1) is a well-known cell cycle-regulated enzyme important for nucleotide metabolism during DNA synthesis [1]. TK1 catalyzes the conversion of thymidine to deoxythymidine monophosphate, which is further phosphorylated to di- and triphosphates preceding its incorporation into DNA. The activity of TK1 is low or absent in resting cells, increasing in the G_1_/S transition and peaking in the S phase, and then disappearing during mitosis [2–5]. Whereas serum TK1 activity is elevated in cancer patients compared with healthy individuals and prognostic in patients with breast and other cancers [6–11], in very few studies have researchers evaluated the utility of serum TK1 for monitoring responses to cancer therapy.

Inhibitors against cyclin-dependent kinase 4/6 (CDK4/6) are an important new class of agents with substantial antitumor activity in patients with advanced hormone receptor-positive (HR+) and human epidermal growth factor receptor 2-negative (HER2−) breast cancer [12–16]. These agents inhibit cell proliferation by activation of retinoblastoma protein, which binds to E2F transcription factors, leading to G_0_/G_1_ arrest [17, 18]. The preferential activity of CDK4/6 inhibitors in luminal or HR+ disease is due to the direct link between estrogen receptor (ER) signaling and CDK4/6 activation, because cyclin D is a direct transcription target of ER and other mitogenic signals associated with endocrine resistance [17, 18].

The potent antiproliferative effect of CDK4/6 inhibition in ER+ breast cancer was demonstrated by tumor Ki-67 analysis in serial biopsies in the NeoPalAna trial (a phase II trial of anastrozole and palbociclib, a CDK4/6 inhibitor, in women with clinical stage II-III ER+/HER2− breast cancer), in which the addition of palbociclib to anastrozole induced complete cell cycle arrest (Ki-67 ≤ 2.7%) in 87% of patients, as compared with 26% following single-agent anastrozole treatment [19]. Because TK1 is a direct E2F transcription target and is strictly cell cycle-regulated, we hypothesized that changes in levels of TK1 activity before and after administration of a CDK4/6 inhibitor would indicate successful inhibition of CDK4/6, and also that serum TK1 activity could serve as a noninvasive surrogate marker of antitumor activity of CDK4/6 inhibition. The serum samples collected before and after palbociclib in the NeoPalAna trial provide an ideal sample set to test this hypothesis. The objectives of this study were to compare TK1 activity in serum collected before and after anastrozole and palbociclib, and to correlate serum TK1 activity with tumor Ki-67 proliferation index and tumor TK1 mRNA levels in patients with early-stage ER+/HER2− breast cancer enrolled in the NeoPalAna trial.

## Methods

### NeoPalAna trial patient population and study procedures

**Fig. 1:**
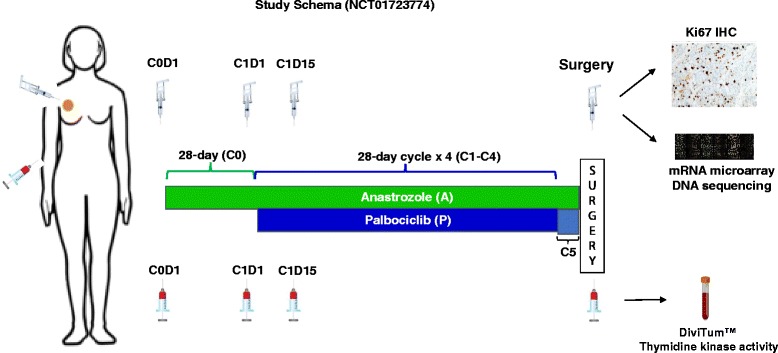
Study schema for the NeoPalAna trial. Eligible patients with clinical stage II-III ER+/HER2− breast cancer received anastrozole monotherapy for 28 days in cycle 0, followed by addition of palbociclib on cycle 1, day 1 (C1D1), for four 28-day cycles unless C1D15 Ki-67 was > 10%. Breast surgery occurred after a washout of 3–5 weeks of palbociclib while the patient was continued on anastrozole, except for eight patients who also received cycle 5 of palbociclib immediately prior to surgery. Serial tumor biopsies and blood collections occurred at baseline, C1D1, C1D15, and time of surgery

The NeoPalAna trial is a neoadjuvant phase II trial of palbociclib, a CDK4/6 inhibitor, and anastrozole for clinical stage II or III ER+/HER2− breast cancer, with the primary endpoint of complete cell cycle arrest (Ki-67 ≤ 2.7%) with the combination of anastrozole and palbociclib. The patient population and trial results were described in our previous publication [19]. As illustrated in Fig. 1, patients received anastrozole 1 mg orally daily for one 28-day cycle (cycle 0, C0), followed by addition of palbociclib 125 mg orally daily (days 1–21 of each 28-day cycle) starting with cycle 1, day 1 (C1D1), for four cycles, unless cycle 1, day 15 (C1D15), tumor Ki-67 was > 10%, in which case patients were taken off study owing to inadequate response. Pre- and perimenopausal women received goserelin 3.6 mg subcutaneously every 28 days. Anastrozole was continued until surgery, occurring 3–5 weeks after palbociclib exposure, except in eight patients for whom an additional 10–12 days of palbociclib (cycle 5) was administered until surgery, following the four cycles of combination therapy. Serial biopsies and blood collections were obtained at baseline (prior to C0), C1D1, C1D15, and time of surgery. Tumor biopsies were centrally analyzed for tumor Ki-67 level using pathologist-guided image analysis [20]. In this trial, we enrolled 50 patients (18 premenopausal and 32 postmenopausal) (Table 1) with a median age of 58 (range 34–79) years, and demonstrated the potent antiproliferative effect of palbociclib in ER+/HER2− breast cancer, even among patients resistant to anastrozole [19]. This trial provided an appropriate sample set to correlate serum TK1 activity with palbociclib treatment and tumor Ki-67 response. TK mRNA levels were derived from microarray analysis of tumor RNA (Agilent Genomics, Santa Clara, CA, USA) [19]. Serum TK1 activity was determined at study enrollment (baseline, C0D1), C1D1, C1D15, and time of surgery.

**Table 1:** Patient characteristics (*n* = 50)

Characteristics	Data
Age, years, median (range)	58 (34–79)
Race	
White	47 (94%)
Black	3 (6%)
Menopausal status	
Premenopausal	18 (36%)
Postmenopausal	32 (64%)
PgR	
Negative	3 (6%)
Positive	47 (94%)
Tumor grade	
1	12 (24%)
2	31 (62%)
3	7 (14%)
Clinical stage	
II	36 (72%)
III	14 (28%)

*PgR -* Progesterone

### DiviTum^TM^ assay for serum TK1 activity measurement

The DiviTum^TM^ assay (Biovica International, Uppsala, Sweden) was used for determination of serum enzymatic activity of TK1 according to the manufacturer’s instructions (http://biovica.com/), as previously described [21]. When serum is mixed with the reaction mixture in a 96-well enzyme-linked immunosorbent assay (ELISA) titer plate, bromodeoxyuridine (BrdU) monophosphate is generated by TK reaction, which is further phosphorylated to BrdU triphosphate and incorporated into a DNA strand bound to the bottom of the well in the microtiter plate. BrdU incorporation is then detected by ELISA using an anti-BrdU monoclonal antibody conjugated to enzyme alkaline phosphatase and a chromogenic substrate, producing the optical density of the color. The absorbance readings to DiviTum units per liter (Du/L) are converted using the values from standards with known TK activity, with a working range from 20 to 4000 Du/L. The analyses were performed at the Biovica laboratory in Uppsala, Sweden, and investigators were blinded to patient or tumor data.

### In vitro cell culture experiment for effect of palbociclib on intracellular TKA

The human cell line K562S (Sigma-Aldrich, St. Louis, MO, USA) was seeded into T25 flasks (3 million cells/flask) containing RPMI 1640 medium (Thermo Fisher Scientific, Waltham, MA, USA) supplemented with 10% FBS (Thermo Fisher Scientific), 100 U/ml penicillin, and 100 U/ml streptomycin (Thermo Fisher Scientific) and treated with palbociclib (0.1 nM to 100 μM; Selleckchem, Houston, TX, USA) for 6 h. Cells were then harvested for determination of cell viability by trypan blue viability assay or lysed for intracellular TK activity by DiviTum assay.

### Statistical analysis

Box plots were generated to demonstrate tumor Ki-67 and TK1 mRNA by time point in all patients. Line plots displayed the levels of serum TK1 activity and Ki-67 by time point in patients in three tumor Ki-67 response categories. The Wilcoxon signed-rank test was used for comparison between time points of serum TK1 activity, tumor Ki-67 index, or tumor TK1 mRNA level. A value of 20 Du/L was used to impute the measurements of TK1 under the detection limit of 20 Du/L for statistical analysis. The subject-level bivariate correlation coefficient (BCC) between serum TK1 and tumor Ki-67 (in logarithmic scale) was calculated using the Bland-Altman method [22], a meta-analysis approach, and the bivariate linear mixed effects model [23]. The concordance of serum TK1 activity change and tumor Ki-67 level change was evaluated by calculating the sensitivity and specificity of decrease in TK1 for predicting decrease in tumor Ki-67 using data at C1D1, C1D15, and time of definitive surgery, excluding the data of the eight patients who were additionally treated with cycle 5. Noncomparable data, such as undetectable TK1 activity at both time points, was also excluded. All tests were two-sided, and significance was set at a 5% α level. All statistical analyses were performed using R version 3.3.2 software (R Foundation for Statistical Computing, Vienna, Austria).

## Results

### Preclinical data indicating CDK4/6 inhibition reduces intracellular TK1 activity in a dose-dependent manner

**Fig. 2:**
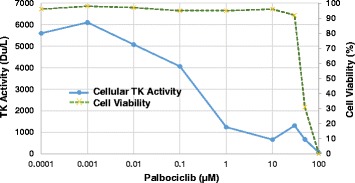
Dose-dependent reduction of thymidine kinase 1 (TK1) activity in response to cyclin-dependent kinase 4/6 inhibition in vitro. K562S cells were treated for 6 h with palbociclib at the indicated concentrations, followed by analysis of cell viability and TK1 activity. TK1 activity was reduced in a linear and dose-dependent manner in response to palbociclib prior to changes in cell viability. *Du/L* DiviTum units per liter

To assess the effect of CDK4/6 inhibition on intracellular TK1 activity, the human cell line K562S was cultured in the presence of increasing concentrations of palbociclib (0.1 nM to 10 μM) for 6 h and harvested for DiviTum analysis. Cell viability was also examined using trypan blue at the same time. As shown in Fig. 2, TK1 activity was reduced in a linear and dose-dependent manner in response to palbociclib with the short duration (6 h) of drug exposure, when the effect on cell viability had not yet become obvious.

### CDK4/6 inhibition reduced serum TK1 activity in NeoPalAna trial

**Fig. 3:**
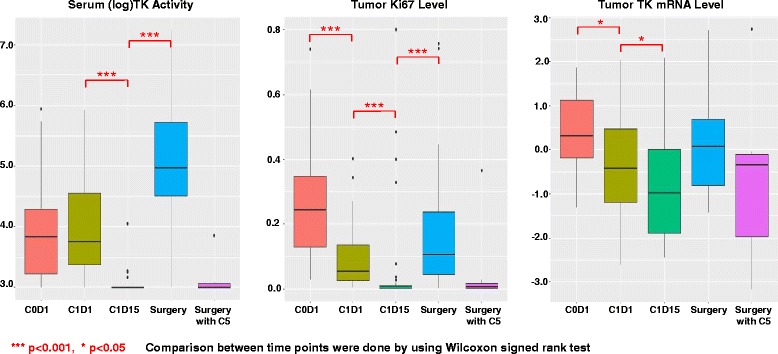
Box plots of serum thymidine kinase 1 (TK1), tumor Ki-67, and tumor TK1 messenger RNA (mRNA) levels by time point for patients enrolled in the NeoPalAna trial. The comparison between time points was done using the Wilcoxon signed-rank test. Similar to tumor Ki-67, serum TK activity was significantly lower on cycle 1, day 15 (C1D15), 2 weeks after adding palbociclib. However, unlike tumor Ki-67 and TK1 mRNA levels, serum TK1 activity did not differ between baseline (C0D1) and cycle 1, day 1 (C1D1), with anastrozole alone. Similar to tumor Ki-67, a significant rebound increase in serum TK1 level was observed at the time of surgery with palbociclib withdrawal, which was suppressed when an additional cycle (cycle 5) of palbociclib was added before surgery. Tumor TK1 mRNA change at surgery time points did not reach statistical significance. *** *p* < 0.001, * *p* < 0.05

To determine whether serum TK1 activity could serve as a surrogate marker for CDK4/6 inhibition and tumor cell proliferation in patients receiving CDK4/6 inhibitors, we analyzed the sample set collected from patients with clinical stage II-III ER+/HER2− breast cancer who received neoadjuvant anastrozole and palbociclib in the NeoPalAna trial [19]. As shown in Fig. 3 and Table 2, there was no statistically significant difference in TK1 activity between baseline and C1D1 following 28 days of anastrozole monotherapy (median serum TK activity was 46 versus 42.55 Du/L, respectively; *p* = 0.52), despite a significant reduction in tumor Ki-67 index, as well as, a reduction in tumor TK mRNA level. In contrast, a striking decline in TK activity was observed 2 weeks after initiation of palbociclib (C1D15), with a median serum TK activity of less than  20 Du/L, (*p* < 0.001); the serum TK activity was below the detection limit of 20 Du/L in 92% (44 of 48) of patients. The remaining four participants had serum TK activity of 24, 26, 26, and 58 Du/L, respectively. This indicates a profound on-target inhibitory effect induced by palbociclib. Following palbociclib withdrawal, the median serum TK level increased significantly from C1D15 to surgery (143.96 Du/L at surgery), indicating recovery of CDK4/6 inhibition, with a similar rebound in tumor Ki-67 observed at the time of surgery. When an additional 10–12 days of palbociclib was given (cycle 5) to eight patients prior to surgery , the serum TK level remained suppressed at the time of surgery at a level similar to C1D15 (median serum TK activity < 20 Du/L; *p* = 0.7893). Collectively, these data indicate that serum TK activity could serve as a pharmacodynamic marker of CDK4/6 inhibition.

**Table 2:** Thymidine kinase 1 activity, Ki-67 and thymidine kinase messenger RNA over time

Time point	Serum TK1		Tumor Ki-67		Tumor TK1 mRNA	
	Median (IQR) (Du/L) (number of cases < 20 Du/L)	No. of participants	Median (IQR) (%)	No. of participants	Median (IQR) (%)	No. of participants
Baseline (C0D1)	46 (25–73) (9 cases < 20 Du/L)	48	24.34% (12.91–34.87%)	45	0.315 (−0.178 to 1.128%)	16
Cycle 1, day 1 (C1D1)	42.55 (29–94.6) (8 cases < 20 Du/L)	49	5.37% (2.52–13.51%)^a^	45	−0.42 (−1.19 to 0.47%)^b^	33
Cycle 1, day 15 (C1D15)	<20 (<20 to < 20)^a^ (44 cases < 20 Du/L)	48	0.78% (0.24–1%)^a^	45	0.98 (−1.89 to 0%)^b^	29
Surgery without C5	143.96 (90.9–306.4)^a^ (3 cases < 20 Du/L)	31	10.63% (4.59–23.67%)^a^	27	0.07 (−0.81 to 0.69%)	17
Surgery with C5	<20 (<20–21.5) (3 cases < 20 Du/L)	6	0.52% (0.16–1.66%)	7	−0.35 (−1.98 to −0.11%)	6

*Abbreviations: Du/L* DiviTum units per liter, *mRNA* Messenger RNA, *TK1* Thymidine kinase 1

C1D1: after 28 days of anastrozole (with goserelin if premenopausal) monotherapy

C1D15: 2 weeks following the addition of daily palbociclib to anastrozole on cycle 1, day 1

Surgery without C5: surgery occurred after four cycles (each cycle is 28 days) of palbociclib plus anastrozole, followed by a washout of palbociclib for 3–5 weeks before surgery

Surgery with C5: cycle 5 (10–12 days of palbociclib) was administered immediately prior to surgery

^a^ *p* < 0.001 compared with the preceding time point using the Wilcoxon signed-rank test (a value of 20 Du/L was used to impute the measurements of TK1 under the detection limit of 20 Du/L)

^b^ *p* < 0.05 compared with the preceding time point using the Wilcoxon signed-rank test (a value of 20 Du/L was used to impute the measurements of TK1 under the detection limit of 20 Du/L)

### Serum TK activity in response to palbociclib by tumor Ki-67 response category

**Fig. 4:**
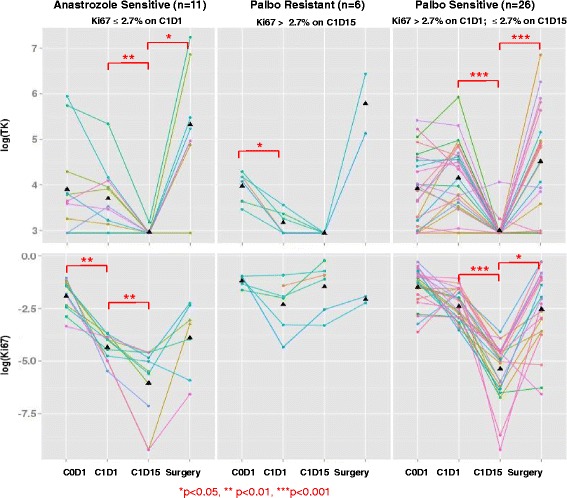
Serum thymidine kinase 1 (TK1) activity and tumor Ki-67 by time point and Ki-67 response category for individual patients. TK (*top panel*) and Ki-67 (*lower panel*) levels in logarithmic scale over time for individual patients are shown in the line graphs. TK activity and Ki-67 from the same patient are denoted by the same line color. Palbociclib (Palbo) significantly reduced serum TK activity 2 weeks after initiation in the anastrozole-sensitive or palbociclib-sensitive group by Ki-67. Palbo washout led to a significant increase in serum TK at the time of surgery. * *p* < 0.05, ** *p* < 0.01, *** *p* < 0.001. Filled triangles denote the average of TK or Ki-67 from different patients at the corresponding time points

To dissect the interaction between serum TK1 activity in tumors with varying responses to palbociclib, we analyzed the TK1 data by tumor Ki-67 response category using the cut-off point of 2.7% (at which level complete cell cycle arrest was defined): anastrozole-sensitive (C1D1 Ki-67 ≤ 2.7%), palbociclib-sensitive (C1D1 Ki-67 > 2.7% but C1D15 Ki-67 ≤ 2.7%), and palbociclib-resistant (C1D15 Ki-67 > 2.7%). As illustrated in Fig. 4, serum TK levels were significantly reduced at C1D15 in both the anastrozole-sensitive and palbociclib-sensitive groups, followed by recovery at surgery due to palbociclib washout. Neither serum TK1 activity nor tumor Ki-67 value significantly changed in the palbociclib-resistant category.

### Concordance in direction of changes between serum TK activity and tumor Ki-67 in response to palbociclib

**Fig. 5:**
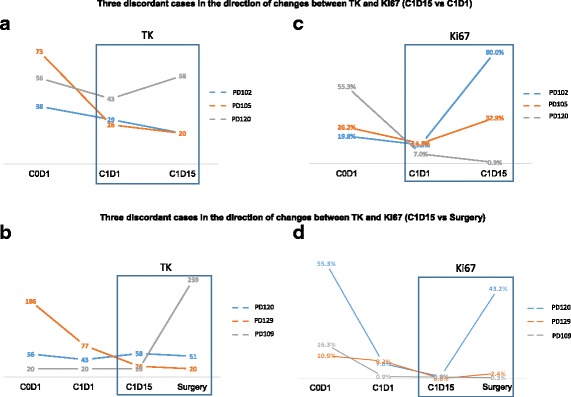
Serum thymidine kinase 1 (TK1) activity and tumor Ki-67 by time point for individual cases discordant in the direction of changes between TK1 and Ki-67 levels. **a** and **b** Line graphs for TK1 activity and Ki-67 levels, respectively, for the three cases discordant when comparing data between cycle 1, day 15 (C1D15), and cycle 1, day 1 (C1D1), time points. **c** and **d** Line graphs for TK1 activity and Ki-67 levels, respectively, for the three cases discordant upon comparing data between C1D15 and surgery time points

To assess palbociclib’s effect and evaluate concordance, data were compared between C1D15 and C1D1, as well as between C1D15 and surgery (no cycle 5), in individual patients. The overall concordance rate in the direction of change between serum TK and tumor Ki-67 by palbociclib was 89.8% (53 of 59 patients, 95% CI 79.2% - 96.2%) (Table 3). The sensitivity and specificity of the decrease in serum TK activity in predicting tumor Ki-67 reduction to palbociclib were 94.1% (32 of 34, 95% CI 86.2% -100%), and 84% (21 of 25, 95% CI 69.6% - 98.4%), respectively. The κ-statistic was 0.76 (*p* < 0.001), indicating substantial agreement between the two tests. The six discordant instances between the effect of palbociclib on tumor Ki-67 and serum TK1 activity are shown in Fig. 5. Two of the three discordant cases between C1D1 and C1D15 time points had serum TK levels in the 20s (with 20 Du/L being the detection limit) at C1D1 (Fig. 5a and b), and one of the three discordant cases between C1D15 and surgery time points had a minimal change in both tumor Ki-67 and serum TK levels. This observation may have limited the comparison between tumor Ki-67 and serum TK activity changes.

**Table 3:** Concordance between changes in serum thymidine kinase and changes in Ki-67 by palbociclib

	Ki-67 ↓ (*N*)	Ki-67 ↑ (*N*)	Total (*N*)
TK ↓ (*n*)	32	4^a^	36
TK ↑ (*n*)	2^a^	21	23
Total (*n*)	34	25	59

*TK*, Thymidine kinase

^a^ Discordant cases

The patient-level BCC between serum TK1 and tumor Ki-67 was 0.46 by both the Bland-Altman method [22] and a bivariate linear mixed effects model [23], indicating a medium correlation between the two. However, the absolute value of serum TK1 activity at C1D15 did not predict the persistent tumor proliferation assessed by Ki-67 staining (Fig. 4 and Additional file 1: Table S1), because all six cases had a serum TK1 activity below the detection limit of 20 Du/L. Because of the significant reduction in serum TK1 levels following anastrozole, with serum TK1 levels < 20 Du/L in three patients and only minimally elevated in three other patients (26, 29, and 35 Du/L, respectively), we were unable to assess the association between changes in serum TK1 activity and Ki-67 between C1D1 and C1D15 time points in the palbociclib-resistant category.

## Discussion

Blood-based biomarkers are of great interest as noninvasive tools for assessing prognosis and in disease monitoring. Circulating tumor cells, exosomes, circulating tumor nucleic acids, and secreted proteins are the most frequently investigated of these potential biomarkers [24]. However, the clinical utility of such biomarkers is often limited by unsatisfactory sensitivity, specificity, and inter-laboratory reproducibility [25]. The DiviTum TK1 activity assay has been demonstrated to be a highly sensitive and reliable measurement tool to measure cell proliferation [6]. Because TK1 is a cell cycle-regulated enzyme that plays a critical role in DNA synthesis, we investigated whether serum TK1 activity could be used as a surrogate marker of the antiproliferative effect of palbociclib in patients with early-stage ER+/HER2− breast cancer enrolled in the NeoPalAna trial (neoadjuvant palbociclib and anastrozole).

This study demonstrates that serum TK1 activity was significantly reduced after 2 weeks of treatment with palbociclib, and that changes in serum TK1 significantly correlated to changes in tumor Ki-67 proliferation index and tumor TK1 mRNA levels. The overall concordance rate in the direction of changes between serum TK and tumor Ki-67 induced by palbociclib was 89.8% (53 of 59, 95% CI 79.2% -96.2%). A reduction in serum TK1 activity by palbociclib had a sensitivity of 94.1% (32 of 34, 95% CI 86.2% -100%) and a specificity of 84% (21 of 25, 95% CI 69.6% -98.4%) in predicting tumor Ki-67 response in this patient population. To our knowledge, this is the first study suggesting that serum TK1 activity may be a promising noninvasive pharmacodynamic marker of the antiproliferative effect of CDK4/6 inhibitors.

Uncontrolled cell proliferation is one of the key hallmarks of cancer [26]. The value of TK1 as a cell proliferation marker was initially explored using IHC to study human breast cancers [27]. Compared with the expression of proliferating cell nuclear antigen (PCNA), although expression of both TK1 and PCNA was significantly higher in malignant than in nonmalignant lesions, only TK1 was associated with tumor stage or histological grade [28], suggesting that it is a better proliferative marker than PCNA. Serum TK1 was subsequently investigated as a tumor marker using monoclonal or polyclonal antibodies against TK1, demonstrating significantly higher levels in preoperative breast cancer patients than in healthy volunteers or patients with benign tumors or following curative surgery for breast cancer [29]. A separate study demonstrated that higher serum TK1 levels 3 months after breast cancer surgery were associated with increased risk of both locoregional and distant recurrence [30]. These earlier studies led to further interest in developing serum TK1 assays and investigation into clinical application. The innovative technology of the DiviTum assay enables the measurement of serum TK1 activity with high sensitivity and is compared favorably with other assay platforms [6]. DiviTum serum TK1 activity has been explored as a prognostic marker in solid tumors, including breast cancer [6–9, 21, 31]. Specifically, in a study of 368 women, including 149 healthy blood donors (control), 59 patients with benign breast disease (BBD), and 160 patients with primary breast cancer, serum TK1 activity was significantly higher in those with invasive breast cancer or with proliferative BBD than in those with nonproliferative BBD and healthy control subjects [6]. Furthermore, serum TK1 activity was significantly associated with tumor size, lack of ER and PgR, tumor grade, and molecular subtype [6]. Multivariate analyses adjusting for stage, grade, and HR status demonstrated that serum TK1 was an independent predictor of disease recurrence (*p* = 0.013) [6]. Additional studies demonstrated that TK1 activity was associated with progression-free survival and overall survival in patients with advanced and metastatic breast cancer [21]. However, in few studies only have researchers investigated the clinical utilityof serum TK1 activity in monitoring therapeutic response to anti-neoplastic agents.

The particular interest in assessing serum TK1 activity in response to CDK4/6 inhibitors stems from the known cell cycle-inhibitory properties of these agents and their importance in the management of patients with advanced HR+ breast cancer [18, 32]. Three CDK4/6 inhibitors, including palbociclib, ribociclib, and abemaciclib, are in clinical development for the treatment of breast cancer and other solid malignancies. All three CDK4/6 inhibitors have received U.S. Food and Drug Administration approval for the treatment of advanced HR+/HER2− breast cancer (Drugs@FDA; https://www.accessdata.fda.gov/scripts/cder/daf/). However, despite this success, especially in endocrine-naive disease, resistance to CDK4/6 inhibitors eventually develops, and a significant proportion of patients with HR+/HER2− breast cancer fail to respond to CDK4/6 inhibitors in the second-line setting (>30%) or beyond (>60%) following progression on endocrine therapy [11, 13, 33]. The clinical application of  early response marker could lead to beneficial changes in determining the optimal therapeutic approach. Thus, the association between changes in serum TK1 activity and tumor Ki-67 response to palbociclib observed in the NeoPalAna trial provides the foundation for future exploration of the potential predictive nature of serum TK1 activity on response and progression-free survival in patients receiving CDK4/6 inhibitors for  metastatic disease.

Our study is limited by its small sample size. However, the study is unique in its ability to obtain concurrent tumor biopsies and serum sample collections for Ki-67 IHC and TK1 activity at serial time points in patients with newly diagnosed, untreated HR+/HER2− breast cancer. The initial treatment with anastrozole monotherapy prior to the addition of palbociclib and the washout of palbociclib prior to surgery allows for the evaluation of dynamic changes in serum TK1 activity.

In this study, serum TK1 activity was not significantly changed following treatment with 28 days of anastrozole (Table 2). This is in spite of the statistically significant reduction in tumor TK1 mRNA at C1D1 (Table 2). In addition, a rise in serum TK1 activity at C1D1 was observed in 3 of the 11 patients in the anastrozole-sensitive group, although the changes were relatively small (from baseline of  < 20, 38, and 44 Du/L to 34, 60, and 50 Du/L on C1D1, respectively) (Fig. 4). This was unexpected because anastrozole inhibits CDK4/6 indirectly through regulating cyclin D1 [34, 35]. As demonstrated in our initial publication of the NeoPalAna trial, anastrozole regulated the mRNA expression levels of a wide range of genes, including those that were further suppressed by the addition of palbociclib [19]. This is supported by the significant reduction in tumor TK1 mRNA following anastrozole at C1D1 and a reduction of serum TK1 activity in ~ 50% of patients. One possible explanation for the lack of significant serum TK1 response following anastrozole is the relatively significant contribution of serum TK1 activity from noncancer cells in the early-stage breast cancer setting. In a study in which investigators compared serum TK1 activity between healthy subjects and patients with breast cancer, the median and IQR value for TK1 activity activity was 16 Du/L (IQR 9–33 Du/L) in the healthy blood donors (*n* = 149) and moderately increased in patients with primary breast cancer prior to surgical exicision (n=160), with median level of 37 Du/L (IQR 20–92 Du/L), respectively. [6]. Although a statistically significant difference in serum TK1 activity was observed between the two groups, overlapping values exist. As shown in Table 2, the median baseline serum TK1 in patients enrolled in the NeoPalAna trial was 46 Du/L (IQR 25–73 Du/L), which is similar to that observed in the previous study [6]. Therefore, the antitumor effect of anastrozole may be difficult to translate into serum TK1 changes, because the antiproliferative effect of anastrozole is restricted to the estrogen-dependent breast cancer cells, whereas the nonmalignant cells are otherwise not affected. This could particularly be the case in patients with lower baseline serum TK1 levels. The slight or moderate rise of serum TK1 activity at C1D1 in those three patients could also reflect fluctuations in individuals because TK1 has a short half-life [4]. Therefore, studies including patients with advanced disease in which tumor cells contribute to the majority of the serum TK1 activity are warranted.

Similarly, the discordance between serum TK1 and tumor Ki-67 change in response to palbociclib, as well as, the low serum TK1 activity despite persistent tumor cell proliferation observed in patients in the palbociclib-resistant category, could be explained by the inhibitory effect of palbociclib on CDK4/6 in both cancer and non-cancer cells in this setting of  early-stage disease. However, we could not rule out the possibility that serum TK1 activity is reduced only in CDK4/6-dependent cancer cells. Larger studies in patients with advanced disease will ultimately provide further insight and address these possibilities.

## Conclusions

Our study provides the first evidence that serum TK1 activity as early as 2 weeks following CDK4/6 inhibitors is highly correlated with tumor cell proliferation response in patients with early-stage HR+ breast cancer. Future studies investigating the value of serum TK1 activity in monitoring treatment response and survival outcomes for patients with metastatic breast cancers treated with endocrine therapy and CDK4/6 inhibitors are therefore warranted.

## Additional file

Additional file 1: Table S1. Serum TK1 activity and tumor Ki67 levels by time point from patients in the palbociclib-resistant category (Ki67 > 2.7% at C1D15) (DOCX 11 kb)

## Abbreviations

BBD: Benign breast disease
BCC: Bivariate correlation coefficient
BrdU: Bromodeoxyuridine
C1D1: Cycle 1, day 1
C1D15: Cycle 1, day 15
CDK: Cyclin-dependent kinase
Du/L: DiviTum units per liter
ELISA: Enzyme-linked immunosorbent assay
ER: Estrogen receptor
HER2−: Human epidermal growth factor receptor 2-negative
HR+: Hormone receptor-positive
HRPO: Human Research Protection Office
IHC: Immunohistochemistry
IQR: Interquartile range
IRB: Institutional review board
mRNA: Messenger RNA
PCNA: Proliferating cell nuclear antigen
PgR: Progesterone
TK1: Thymidine kinase 1
UAB: University of Alabama at Birmingham
