# Supplementary material for: Serum thymidine kinase 1 activity as a pharmacodynamic marker of cyclin-dependent kinase 4/6 inhibition in patients with early-stage breast cancer receiving neoadjuvant palbociclib
Source: Breast Cancer Res. 2017 Nov 21;19:123. doi: 10.1186/s13058-017-0913-7 (PMC5699111; doi:10.1186/s13058-017-0913-7)
Supplement: Additional file 1: Table S1. — Serum TK1 activity and tumor Ki67 levels by time point from patients in the palbociclib-resistant category (Ki67 > 2.7% at C1D15) (DOCX 11 kb) [file 13058_2017_913_MOESM1_ESM.docx]

| **Table S1 Serum TK1 Activity and Tumor Ki67 Levels by Time point from Patients in the Palbociclib Resistant Category (Ki67 > 2.7% at C1D15)** | | | | | | |
| --- | --- | --- | --- | --- | --- | --- |
|  | **Serum TK1 Activity (Du/L)** | | | **Tumor Ki67 (%)** | | |
| **Patient ID** | **Baseline** | **C1D1** | **C1D15** | **Baseline** | **C1D1** | **C1D15** |
| PD102 | 38 | 29 | <20 | 19.8% | 13.5% | 80.0% |
| PD106 | 32 | <20 | <20 | 38.0% | 40.2% | 48.4% |
| PD205 | 65 | <20 | <20 | NA | 24.1% | 40.0% |
| PD105 | 73 | 26 | <20 | 26.2% | 14.5% | 32.9% |
| PD113 | 59 | <20 | <20 | 37.8% | 1.3% | 7.8% |
| PD108 | 65 | 35 | <20 | 33.7% | 3.8% | 3.7% |
